# What influences uptake of psychosocial interventions by people living with early dementia? A qualitative study

**DOI:** 10.1177/14713012211007397

**Published:** 2021-05-06

**Authors:** Becky Field, Elizabeth Coates, Gail Mountain

**Affiliations:** School of Health and Related Research (ScHARR), 7315 University of Sheffield, UK; Centre for Applied Dementia Research, University of Bradford, UK; School of Health and Related Research (ScHARR), 7315 University of Sheffield, UK

**Keywords:** dementia, psychosocial interventions, non-pharmacological interventions, early dementia, post-diagnostic support, uptake

## Abstract

**Background:**

Health policy promotes post-diagnostic support for people affected by dementia. Evidence suggests psychosocial interventions can effectively support people living with dementia after diagnosis. Yet, what influences uptake of psychosocial interventions by people with early dementia is poorly understood. This research aimed to identify influences on uptake of psychosocial interventions by people with early dementia.

**Methods:**

Sixteen face-to-face semi-structured interviews with people with early dementia, either alone or with a family member(s), were completed. Twelve staff participated in semi-structured interviews or a focus group. Thematic analysis and triangulation enabled identification of overall themes across different participant groups and interview types.

**Main Findings:**

Four overarching themes influencing uptake were identified: (1) adjusting to a diagnosis, (2) appeal of activities and perception of benefit, (3) service and societal context, and (4) relationships and communication. Individual responses to diagnosis, experiences of dementia and dementia services influenced uptake. Group interventions were discussed the most by all participants. Group interventions offering social contact, peer support, information, enjoyable activities and mental stimulation were valued. However, group interventions specifically aimed at people with dementia did not appeal to all. Ability to travel and convenience of locations were important. Continuing with community activities not focused on dementia was valued. Stigma around dementia appeared to discourage uptake. Emotional and practical support from family was key to facilitating uptake as were the relationships between people with dementia and staff.

**Conclusion:**

A complex interplay of individual, service and societal influences affect uptake of psychosocial interventions by people with early dementia. How interventions and which services can enable people with early dementia remain engaged in their everyday lives needs consideration. Further research examining uptake of specific interventions commonly offered to people living with early dementia is needed. Involving people with early dementia in designing interventions aiming to support them is paramount.

## Introduction

The importance of providing timely and appropriate treatment and support to people after a diagnosis of dementia is recognised nationally and internationally (Department of Health, 2009, 2012, 2015; Global Action Against Dementia, 2013; Hodge et al., 2016; NHS England, 2017). In England, NHS memory services aim to facilitate timely diagnosis and post-diagnostic support. The Memory Services National Accreditation Programme (MSNAP) (Hodge et al., 2016) and the National Institute for Health and Care Excellence (NICE, 2018) recommend psychosocial interventions (hereafter referred to as interventions) for people living with dementia and family members after diagnosis. Such interventions include cognitive stimulation therapy (CST), life story work, cognitive rehabilitation, psychology and occupational therapy (Hodge et al., 2016). Evidence suggests interventions can support people with dementia and family members after diagnosis by improving quality of life, cognition and functional abilities (e.g. Keogh et al*.*, 2019; Olazarán et al*.*, 2010; Oyebode & Parveen, 2016; McDermott et al*.*, 2018). Much of this evidence is from studies including people with early or mild dementia and those with moderate dementia or levels of cognitive impairment (e.g. Clare et al., 2019; Graff et al., 2006; Spector et al., 2003). Whilst how intervention studies define early, mild or moderate dementia varies (Keogh et al., 2019), some evidence indicates people with early dementia specifically can benefit from interventions (e.g. Leung et al., 2014; Martin et al., 2015; Quinn et al., 2016; Sprange et al., 2016).

To benefit from what interventions can offer, people with dementia need to accept intervention offers initially. Experience of offering an occupational therapy intervention, as part of a research study, led to the authors’ interest in this topic as many potentially eligible people declined the intervention as it appeared some did not consider it suitable for them (Field et al., 2019b). There appears to be limited research explicitly focused on uptake of interventions by people with early dementia. Intervention studies tend to focus on reporting effectiveness and outcomes of interventions, generally reporting numbers for non-participation, but often giving no further explanation (e.g. Graff et al., 2006; Streater et al., 2016) or only limited explanation. For example, a few people with dementia and carers were reported as declining cognitive rehabilitation because they were content with their current situation (Clare et al., 2019), and carers were reported as being too stressed to participate in a carer-delivered CST intervention (Milders et al., 2013). Some qualitative studies examining feasibility or intervention acceptability suggest that interventions perceived as meeting needs or preferences of people with dementia and carers facilitate acceptance (Quinn et al., 2016; Sprange et al., 2015). Field et al. (2019a) conducted a secondary qualitative analysis aiming explicitly to identify influences on uptake of an occupational therapy intervention, offered as part of a research study. However, influences on uptake of interventions and reasons for declining research studies may differ from those for interventions offered in practice in local dementia services.

Research examining service use by people with dementia also exists (e.g. Górska et al., 2013; Gilbert et al., 2017; Griffith et al., 2016; Innes et al., 2014) but has not focused specifically on uptake of interventions, nor those with early dementia. Also, often service use studies have focused on carer perspectives and/or those with more moderate or severe dementia (Lloyd & Stirling, 2011; Stephan et al., 2018) or pre-diagnosis help seeking (Begum et al., 2013; Chrisp et al., 2012).

Existing evidence thus offers limited understanding about what affects uptake of interventions in practice, offered to support people with early dementia in the United Kingdom. This study aimed to contribute to this evidence gap.

## Study Aims

The aim of this study was to explore and examine influences on the uptake of psychosocial interventions by people with early dementia after diagnosis.

## Methods

### Sampling and recruitment

A convenience sample (Ritchie et al., 2014) of people with dementia aged over 65 years and staff willing and able to be interviewed within the time frame available were recruited in England via two NHS memory services in two different local authority areas, a local branch of the Alzheimer’s Society and the ‘Join Dementia Research’ (https://www.joindementiaresearch.nihr.ac.uk) research register.

People with dementia and family members were included because we wanted to give people with dementia the option of having a family member join them for interview or not, as other researchers have done (Innes et al., 2014; Nygård, 2006). Giving this choice aimed to facilitate participation of people with dementia who wanted the support of another person whilst allowing those who did not want this, or were without a suitable family member, to participate alone. People with dementia experiencing memory or communication difficulties or reduced self-confidence may feel they need support to participate in an interview and other people can support the person with dementia and act as informants alongside the main participant (Nygård, 2006).

Including staff aimed to illuminate the contexts within which interventions are offered by gaining the perspectives of those who offer and provide interventions. In addition, we considered it unlikely those who declined interventions would be recruited, so asking staff to discuss their experience of people declining interventions was worthwhile given various types of knowledge can be used to obtain a through and in-depth understanding of a phenomenon (Lambert and Loiselle, 2008). Multiple data sources such as different respondent groups (Denzin, 2009) aid a multidimensional understanding (Farmer et al., 2006) and increase the likelihood that findings will be credible and dependable (Lincoln and Guba, 1985).

Table 1 shows how suitability to participate was established.

**Table 1.** Participant eligibility criteria.

People with dementia	Family member	Staff
Be diagnosed with dementia within the last 2 years	Be the person whom the person with dementia wishes to participate in a joint interview with them, if the person with dementia wishes for a joint interview	Be working in or have worked in dementia services in the NHS or other services; be willing and able to participate in an interview or focus group
Be living with early dementia (self-reported or reported by family carer or staff)		
65 years old or over	18 years old or over	
Be willing and able to participate in an interview and have capacity to consent to the study	Be willing and able to participate in an interview and have capacity to consent to the study	
Be living in the community in their own home or sheltered housing (but not residential or nursing care)		

The rationale for focusing on people with early dementia specifically was because we wanted to identify people whose experience of dementia at the time of interview was such that they would potentially benefit from participating in interventions aimed at people with mild–moderate dementia (e.g. Clare et al., 2019; Graff et al., 2006; Spector et al., 2003) and be able to consent and participate in an interview.

### Data collection: people with dementia and family carers

Face-to-face semi-structured interviews were completed with people with dementia alone or jointly with a family member depending on the person with dementia’s preference. An indicative topic guide based on previous work (Field et al., 2019a, 2019b) supported discussion about:

1. experience of services since diagnosis
2. influences on acceptance or rejection of interventions people with dementia had been offered
3. types of support or interventions participants might wish for

People with dementia were supported to participate in interviews using strategies suggested by McKeown et al. (2010), Murphy et al. (2015) and Novek & Wilkinson (2019). For example, building in time to chat to establish rapport, one-page summaries posted in advance and the researcher identified interventions available in participants’ local areas to help facilitate discussion about potentially familiar interventions. Verbal and written prompts and photographs of memory services and staff were used to aid discussion if needed.

### Data collection: staff

Semi-structured interviews by telephone or face-to-face, depending on preference and one focus group, were held. An indicative topic guide informed by previous work (Field et al., 2019a, 2019b) included:

1. experience of referring to or providing psychosocial interventions
2. influences on people with dementia’s uptake or rejection of interventions
3. types of support or interventions staff might consider appropriate

All interviews were audio-recorded, professionally transcribed and checked for accuracy, except an initial telephone interview with a memory service manager for which handwritten notes were made.

### Ethical considerations

Written informed consent was obtained for all participants. For people with dementia, a capacity assessment screening tool helped establish key components of a person’s capacity to make a decision about participating in this study, according to the Mental Capacity Act (2005). A model of ongoing consent (Dewing, 2007) guided the consent process at each contact with people with dementia. Ethical approval was obtained from an NHS Research Ethics Committee (Reference:17/NW/0414).

### Data analysis

Interview transcripts were analysed using thematic analysis (Braun & Clarke, 2006, 2014; Clarke & Braun, 2017). This involved six phases, summarised in Table 2.

**Table 2.** Phases of thematic analysis and how they were applied in this study.

Phase of thematic analysis (Braun & Clarke, 2006)		How this was applied in this study
Phase 1	Familiarisation	- Each transcript read several times
		- Notes made summarising content and ideas for initial codes
Phase 2	Generating initial codes	- List of initial codes produced applied to each transcript, list edited iteratively until all relevant data coded
Phase 3	Searching for themes	- Codes grouped into potential themes
		- Coded extracts collated into groups of related codes; placed in tables of potential themes.
		- ‘Mind maps’ used to help identify potential themes (codes grouped into clusters of related codes, lines drawn between them to consider relationships between codes)
Phase 4	Reviewing themes	- Groupings of codes and themes reconsidered and adjusted to identify key and subthemes
Phase 5	Defining and naming themes	- Essence of each theme described with a few sentences
		- Each key theme and subtheme named
Phase 6	Reporting	- Key and subthemes reported

The solo interviews with people with dementia and joint interviews were analysed as separate datasets initially. Then, both these types of interview were combined into one dataset for further analysis. Each staff interview and the focus group were also combined to form another, second, dataset. This was because codes initially identified from solo and joint interviews with people with dementia were very similar, as were codes generated from the different kinds of staff interview and focus group. Key themes and subthemes for each separate dataset were identified (see Supplemental material). Cases which did not fit into overall themes were also identified (Spencer et al., 2014; Silverman, 2010).

A triangulation exercise (Farmer et al., 2006) identified similarities and differences between key themes and subthemes from the separate datasets (i.e. solo and joint interviews with people with dementia and staff interviews and focus group). Each dataset was assessed for convergence and difference with the other by re-examining each transcript to identify whether or not it contained data relating to subthemes or key themes identified in the other dataset. This process enabled identification of the overarching themes presented in this article.

Reflexivity was incorporated by recording field notes after all interviews and the focus group. Instances where people with dementia and family members expressed different views and researcher reflections about how each person expressed himself or herself within a joint interview were made to try to ensure perspectives of people with dementia were represented. Reflections were transcribed and coded during analysis to help interpretation. One co-author coded a proportion of the transcripts to aid credibility and trustworthiness of findings. Thematic analysis and triangulation was completed by the first author, and findings were regularly discussed with co-authors.

NVivo software was used to store, organise and support analysis of the anonymised data.

### Description of participants

#### People with dementia and family members

Sixteen people with dementia (aged 66–87 years) were interviewed. Four were interviewed alone, and 12 jointly with one or more family member (aged 58–80 years). Length of interviews ranged from 34 to 80 min. Fifteen interviews took place in participants’ own homes and one in a family carer’s home. Table 3 summarises the main characteristics of people with dementia and family members.

**Table 3.** Main characteristics of people with dementia and family members.

Person with dementia pseudonym	Family member pseudonym	Type of relationship	Type of dementia	Time since diagnosis^a^	Age of person with dementia	Family carer age/s	Living situation
Joint interviews							
Tom	Sally	Partners	AD	Approx. 2 years	81	69	Lived together
Edith	Liz and Colin	Daughter-in-law and son	AD	10 months	87	62 and 64	Lived alone (sheltered accommodation)
Pam	Dave	Wife and husband	FTD	Approx. 2 years	66	64	Lived together
June	Sarah	Mum and daughter	AD	11 months	78	58	Lived with daughter
Steve	Jan	Husband and wife	AD	Within 12 months	70	70	Lived together
Dot	Jenny	Friends	Mixed AD and VD	Within 12 months	84	62	Lived alone
Mavis	Maureen	Sisters	Mixed AD and VD	21 months	87	^b^	Lived with daughter
Larry	Irene	Husband and wife	VD	18 months	77	70	Lived together
George	Linda	Husband and wife	AD	4 months	73	72	Lived together
Jimmy	Aida and John	Husband and wife and son-in-law	AD	14 months	75	77 and 57	Lived with wife
Kathryn	Phillip	Wife and husband	AD	13 months	80	80	Lived together
Iris	Len and Pauline	Wife and husband and daughter	Mixed AD and VD	5 months	74	^b^	Lived together
Solo interviews							
Keith	-	-	‘mixed type’	Approx. 1 year	72	-	Lived alone
Angela	-	-	AD	Approx. 2–3 months	70	-	Lived alone
Beryl	-	-	AD	6 months	81	-	Lived alone
Sue	-	-	AD	Within 12 months	80	-	Lived alone

AD: Alzheimer’s disease; FTD: frontal temporal dementia; VD: vascular dementia.

^a^ Times since diagnosis were reported by participants, where an approximate time is given. This is because participants were unable to recall an exact date or length of time.

^b^ Missing data.

#### Description of staff participants

Twelve staff participated in a focus group or face-to-face or telephone interviews. Interviews were conducted at a participant’s home, participant’s office or at a University. The focus group took place at a memory services building. Length of staff interviews ranged from 30 to 77 min. The focus group lasted an hour. Table 4 presents the types and number of staff participants and data collection method used.

**Table 4.** Types and number of staff and data collection method.

Type of staff participants (and number of each type interviewed)	Method
Clinical psychologist (*n* = 1) and occupational therapist (*n* = 1)	Individual face-to-face
Alzheimer’s Society staff (1 manager and 1 support worker)	Paired face-to-face
NHS memory service manager (*n* = 1)^a^ and doctor (*n* = 1)	Individual telephone
Nurses (*n* = 4), occupational therapists (*n* = 2), support worker (*n* = 1) and manager (*n* = 1). These staff all worked together at one NHS memory service	Focus group

^a^ This manager was interviewed twice; first to aid study planning and again to seek their perspective on questions in the staff topic guide.

## Findings

### Interventions described

All but two participants with dementia described participating in at least one intervention. One person with dementia (June) had so far declined all intervention offers, and another (Steve) had not attended an intervention at the time of interview, but said he was planning to attend the CST group he had been invited to. Amongst both people with dementia and family members and staff, group interventions and CST particularly were the most talked about. No people with dementia or family members reported the person with dementia being offered a personalised intervention. One memory service had a dedicated team to run CST groups. The other memory service also ran CST groups, as well as peer support and education and information groups. Only the psychologist and occupational therapists mentioned offering personalised interventions, such as cognitive rehabilitation. Alzheimer’s Society staff talked about offering individualised telephone support or home visits, depending on personal need. Box 1 summarises the interventions described by people with dementia and family members.

**Box 1 Interventions described by people with dementia and family members.**

1. Group CST and group maintenance CST
2. Group education and information sessions
3. An exercise group
4. A group about being diagnosed and living with dementia
5. A Life Story group
6. Memory cafes
7. Singing for the Brain
8. Home visit/s from a member of Alzheimer Society staff

### Overarching themes

Four overarching themes and seven subthemes about influences on uptake by people with early dementia were identified from all the different interviews and focus group. These are summarised in Table 5.

**Table 5.** Overarching themes and subthemes.

Overarching themes	Subthemes
Adjusting to a diagnosis	Awareness of changes or challenges
Intervention appeal and perceived benefit	Group interventions not appealing
Service and societal context	Scheduled appointments and ‘information overload’
	Resource management within dementia services
	Access and practicalities
	Stigma
Relationships and communication	Pivotal role of family members
	Staff and family members supporting people with dementia manage feelings of fear and anxiety
	Respecting personal choice and being directive

### Theme 1: Adjusting to a diagnosis

This theme is about the process of adjustment after diagnosis and how this seemed to encourage or discourage uptake. Several people with dementia and family members described still coming to terms with the dementia diagnosis. Feelings of shock, fear and distress were expressed by some, as the following quote from Angela illustrates:

‘…. at first I wanted to kill myself. Because I couldn’t see a future…. Being good at what I do that’s really important to me, and suddenly I’ve got this label and I just, I just thought I’d rather be dead..’ Angela (living with dementia)

However, such feelings did not appear to have stopped Angela and others from trying the interventions, such as CST or Singing for the Brain, and most were keen to engage, wanting information, support from others or mental stimulation.

Similarly, staff recognised how people with dementia and families needed time to adjust to the diagnosis. Several talked about how in their experience, struggling to adjust or needing time to get used to the diagnosis could lead to interventions such as CST or education groups being declined.

### Subtheme: Awareness of changes or challenges

Most people with dementia openly acknowledged their diagnosis, describing changes such as memory loss, low mood and frustration. Most of these people were keen to attend interventions given these challenges. However, Angela, Beryl, Sue and George said they did not really feel any different or found it hard to truly believe they had dementia, even though they had been told this was the case by professionals or family. The following quote from George illustrates this:

‘..I don’t really feel any different… some days I do get more forgetful, but I don’t think I suffer so much from that do I?.... You’re the one that notices this more than me…’ George (living with dementia)

Whilst such feelings or beliefs had not prevented these people with dementia accepting interventions, it appeared they might have been encouraged by relatives or staff to engage to try an intervention, rather than believing themselves they would benefit or needed support.

Awareness was also a factor that some staff felt may encourage uptake. For example, the Alzheimer’s Society staff, nurses in the focus group, the occupational therapist and a memory services manager described working with people who did not believe themselves to have dementia or described the impact of dementia on themselves as minimal. These staff felt such people were likely to reject interventions as they did not perceive a need for such support. The following quote from the focus group illustrates this:

‘I think a lot of people don’t think they have dementia and even when they’ve been to the consultant and they’ve had a diagnosis and they come for a post-diagnosis appointment, they still don’t believe they have any form of dementia (Nurse 1)…

… or memory problems (Occupational Therapist)…

….yeah, so tell them to go along to a group for somebody with a memory problem, “well I don’t have one so I wouldn’t need that group”’ (Nurse 1) (Focus group)

### Theme 2: Intervention appeal and perceived benefit

This theme is about the appeal of activities offered and whether people thought this might benefit them. Most of the people with dementia seemed keen to try interventions offered but some were uncertain about whether interventions appealed to them or held potential benefit.

Groups offering opportunities to socialise and peer support were valued by most. Most people with dementia talked a lot about their personal interests and pastimes. Many had been active in retirement and were keen to remain involved via, for example, churches or pensioner’s clubs. These were not activities or groups aimed at people with dementia specifically. Continuing with existing, community-based activities and roles such as looking after grandchildren, seeing family and friends and having holidays or day trips was very important to people. In contrast, staff did not discuss people’s individual interests but did acknowledge that group interventions did not appeal to all as summarised by subtheme ‘group interventions not appealing’. Also, for some of people with dementia, participating in group interventions seemed acceptable perhaps because they were used to being in groups and the activities offered seemed to ‘fit’ their interests and personal narratives. Specific group intervention activities appealed to some people with dementia, such as singing, dancing or playing games. The appeal of such activities appeared related to long-standing interests or hobbies or because people recognised the benefits of being stimulated, as the following quotes illustrate:

‘...I go to one [a group] where I dance...Fred Astaire and Ginger haven’t got a patch on us...I used to be dancing nearly every night when I were young…they said…last time, it’s a veleta, I’m not doing veleta steps, but anyway we got through it’ (Edith, living with dementia)

‘We knew Edith would…want to go to anything she could really…she used to be, as a younger person she, you were quite active in things like WI and all that weren’t you… then there’s the Singing for the Brain. We knew she’d like that because she used to be in choirs…’ Liz (Edith’s daughter-in-law)

‘Well if you look at this one [a memory café], it’s not just a coffee morning…I go every Monday...they’ve got facilities like what the others, the cafes, don’t have. They have billiards, they have games, I play chess, everything to stimulate your mind’ (Keith, living with dementia)

### Subtheme: Group interventions not appealing

However, group interventions did not appeal to all the people with dementia, and some of those who had participated in interventions described feeling reticent about their attending. This is illustrated by Steve explaining his concern about attending a CST group he had been invited to:

‘.... I’m more into doing things, not sitting down and writing or drawing or whatever. I’m sort of a one to one person not sort of sit in a group... brain’s going downhill any rate so it’s not very good. I’m more interested in doing things than actually talking about things’ (Steve, living with dementia)

Further, some people with dementia and a few family members expressed concern about meeting others with dementia because of feeling uncomfortable, perhaps fearful of meeting those more severely affected than themselves or not wanting to share their experiences of dementia with others. In the following quotes Beryl, who wanted to meet a few more people, and June, who had declined to participate in an education group or CST, explain their reticence:

‘…I don’t want to meet lots of people probably…I know there’s a walking group within the Alzheimer’s [Society] but I don’t know really about that…how far down the line they would be with their Alzheimer’s? I’d want to be able to go and just converse with somebody who’s able to, you know’ (Beryl, living with dementia)

‘... It’s all sitting round, all having to talk about what they feel because I think it’s personal to yourself and I don’t think it should be voiced on the stage…It’s as if you’ve got a bad marriage, you wouldn’t like to sit in a group talking about what your husband does and what. I just think it’s personal… Everybody don’t feel the same if they’re losing their sight or losing their memory… If there were suddenly a couple in here, going through same thing, I would be willing to sit and discuss it. But I don’t want a wider audience’ (June, living with dementia)

Several staff also discussed how some people rejected interventions such as CST or education groups because they did not like groups and felt this response should be respected, as illustrated by the following quote:

‘I think the biggest factor we haven’t mentioned in attending groups is people’s personalities…some people just don’t like mixing within a group setting so...[murmurs of agreement from the group ‘yeah yeah’] I think that’s probably the biggest thing that I find, that people say “oh I’ve never been a mixer, I don’t want to do anything like that...[another participant agreeing: ‘yeah yeah’] ... you just have to accept that, if that’s how somebody feels’ Nurse 1 (Focus group)

The psychologist and the focus group agreed that people with dementia and families could be anxious about mixing with other people with dementia, which could discourage uptake of CST.

‘I’ve experienced in groups where somebody’s not been so far along with the dementia where somebody’s come to the group…you can see the anxiety on people and you can see them actually thinking “am I gonna be like that..?” and it actually puts them off coming to groups’ Support worker (Focus group)

Also, some people with dementia and family members talked about declining groups because people they were busy. For example, Dave and Pam regularly cared for grandchildren; Tom said he had jobs to do at home. The focus group also acknowledged similar issues, agreeing that some people with dementia appeared to feel busy or coping with life independently and already felt connected socially. These staff considered that such people perceived little benefit in attending CST groups.

### Theme 3: Service and societal context

This theme is about how the context of services influenced uptake.

#### Subtheme: Scheduled appointments and ‘information overload’

The time constraints of scheduled appointments for people with dementia after diagnosis were highlighted by both staff and some joint interviews with people with dementia and family members, mainly from the family member perspectives. The focus group and the doctor discussed the amount of information they needed to cover, which often felt too much for the person with dementia and carer to process. Both explained how they provided information packs about support services and intervention groups:

‘…we’re aware that a clinic appointment can be very overwhelming, it can appear like white noise, you know they can hear a diagnosis and “I might have to stop driving” and that’s all they get so it’s often helpful for them to digest that information and also circulate it round family and our contact details…’ Nurse 3 (Focus group)

One family member described appointments, in which support was discussed, feeling overwhelming:

‘…at the memory clinic they overwhelm you with information and invite you to all these things like you could be there every day of the week… they tell you about all these workshops and oh I can’t even think about what there were … I think they throw everything at you, in less than an hour or something, and it’s just variable what sticks or what goes in…’ (Linda, wife)

#### Subtheme: Resource management within dementia services

The impact of resource management on the kinds of interventions offered and thus uptake was discussed by both managers interviewed, the Alzheimer’s Society support worker and the psychologist. In contrast to the staff interviews, people with dementia did not discuss resources affecting interventions offered but a few family members did. The impact of the wider context is highlighted by the following quote:

‘...the push has been around increasing diagnosis rates...there’s now a 6 week target to diagnose…So all the resources get invested there…the way that the service is measured…outcomes to the commissioners is on how many people we’re getting diagnosed, not on what happens afterwards.. So whilst that’s been driving it…we’ve been saying…what about when people do get diagnosed what are we offering that’s of any benefit?...’ (Psychologist)

#### Subtheme: Access and practicalities

All accounts indicated the vital importance of convenient transport to enable people with dementia to attend interventions. Some people with dementia were unable to travel independently and so depended on family for travel to interventions.

Some family members talked about their other responsibilities or their own ill health impacting on their ability to take a person with dementia to a group session, consistently or at all. Many staff also felt the absence of transport to interventions limited uptake. The focus group agreed the effort and stress associated with organising and carrying out a journey, or simply the thought of it, could discourage uptake of CST, or that some people with dementia worried about burdening family and so declined. Poor public transport provision within large geographical catchment areas covered by memory services was also noted as sometimes leading to rejection of interventions. How such practical issues limit uptake are highlighted by the following quotes:

‘…[ we] don’t provide transport…that can cause anxiety you know and it does depend whether someone’s got a carer that can actually bring them along’ Occupational Therapist (Focus group)

‘...if people are not physically able to get out of the house that’s going to be obviously an issue, and get transport. There’s no transport to those psychosocial interventions that’s provided. That’s quite a major deal I would say, if there was transport maybe more people would go’ (Doctor)

‘Well I couldn’t go on my own [to a CST group] because I can’t drive (Kathryn, living with dementia) …

… You’d get a bus dear (Phillip, husband)

…Oh no...(Kathryn)

…If there was something this end of town we probably would love it’ (Phillip)

#### Subtheme: Stigma

Societal stigma associated with dementia was highlighted as a barrier to uptake by some staff. The following quote illustrates this concern:

‘…they’ll say “we don’t want to be with other people with Alzheimer’s and we haven’t told anybody that you know my wife’s got Alzheimer’s…we don’t want people knowing that she’s got it so we don’t want to be going to places like that”…You know but there is still a lot of people who do feel that there is this stigma attached to that diagnosis’ Manager (memory services)

In contrast, none of the people with dementia and family members explicitly used the term stigma. However, some talked about responses of friends or family to the diagnosis. Such accounts suggested stigma was certainly part of some people’s experience. For some, this perhaps influenced subsequent uptake of interventions. Stigma may also have contributed to people’s uncertainty about attending group interventions or preferences for pursuing non-dementia activities as outlined in Theme 2 and the subtheme ‘group interventions not appealing’. George and his wife talked about how they had not told friends and family and that George did not like going to memory services, where intervention groups took place. He worried former colleagues who worked nearby may see him:

‘...he’s one of my old work colleagues and if he gets a whiff of I’ve got Alzheimer’s then...I’m sure that it would spread and it would get back to my old work colleagues which I don’t want’ (George, living with dementia)

### Theme 4: Relationships and communication

This theme is about the importance of relationships between the people with dementia and their family members and staff and how these relationships were important influences on uptake.

#### Subtheme: Pivotal role of family members

Family members provided emotional and practical support, in addition to support with transport, which positively influenced uptake. Some provided physical assistance or verbal prompting to support people with dementia get ready to attend groups. Family members offering reassurance or encouragement appeared key when a person with dementia had not been keen or was unsure about trying an intervention. Reflecting this, staff also discussed how they encouraged people with dementia to bring a family member with them to a new group, to offer reassurance. Sue explains the role her son played in facilitating her attendance at a CST group, in the following quote:

‘…I never thought about it because it was [son] that pushed me…I’m really glad I go now because it is nice…He’s bossy like his father; “oh mother come on you don’t want to sit in house all day” …,“I’ll go with you”, and he does and he comes in [into the CST group] now. Because a lot of them go with their husbands you know and we have a right laugh, we do have a right laugh…’ (Sue, living with dementia)

Several people with dementia and family members talked positively about how some staff had communicated with them, and a few talked about negative experiences of staff communication. Positive interactions seemed to influence how these people felt about engaging with the service offering interventions.

#### Subtheme: Supporting people to manage feelings of fear and anxiety

As Theme 1 (adjusting to a diagnosis) indicated, several people with dementia expressed feelings of fear or anxiety whilst adjusting to the diagnosis. This subtheme is about how both family members and staff tried to support people manage such feelings and potentially encouraged uptake. Both family members and staff explained how they tried to reduce worry or anxiety experienced by people with dementia by offering reassurance. The following interaction illustrates how important John’s reassurance and support was to Jimmy and the way a member of staff communicated with Jimmy, helping reassure him further, when Jimmy had been invited to a CST group:

‘*(*crying) *I didn’t want to go into a home* ***…***’ (Jimmy, living with dementia)

…we had to ask [staff name] if [they] could talk to Dad…explain…that we weren’t taking him to keep him, it was for an assessment to see if the courses and stuff were going to help…after… [name] spoke to him…he knew he wasn’t staying, so he were like from…shuffling his feet…to a proper spring in his step …when we said about going back the next time, couldn’t get him back in the car quick enough… But the first one it, he honestly thought that we were locking him up…’ (John, son-in-law)…

Yeah because that’s the only reason that they are wanting me to go to these places, is to assess me and put me away. My John says there’s no way you are going to be locked away’ (Jimmy)

#### Subtheme: Respecting personal choice and being directive

Joint interviews, staff interviews and the focus group highlighted how some family members took a directive approach with their loved one, if they felt the person would benefit from an intervention, or at least trying it. However, staff felt they needed to respect the personal choice of the person with dementia. For example, a few family members (such as George’s wife, Pam’s husband and Jimmy’s son-in-law) described sometimes being directive and persuading people with dementia to try an intervention even if they were not keen. It seemed these family members took this approach based on their relationship with the person with dementia, when they thought interventions might be of benefit and the person would enjoy themselves when they got there. This issue was illustrated by June and Sarah. Sarah felt her mother enjoyed and benefitted from company, and June agreed she liked talking to people but was mostly housebound when Sarah was at work, yet both had described June declining offers of groups so far.

‘I’m going to insist what she does now. Rather than leave it to Mum to decide. I’m going to put things in place so that she’s got no choice…’ (Sarah, daughter)

‘…I don’t mind, if it were anybody else but her I’d say bugger off I’m not going….’ (June, living with dementia)

Staff accounts discussed how some people needed time to adjust and come to terms with the diagnosis and they needed to respect this, but also give people time to consider or adjust, as highlighted in the focus group:

[Researcher: *What if someone sort of says ‘no, don’t want to do the CST group, what might you do in that situation?*]

‘Nothing!’ (Nurse 1)

‘Nothing, it’s their choice’ (Occupational therapist 1)

‘We’d probably raise it again...we do have uptake, it’s not always just at PDS [post diagnostic appointment] is it?’ (Nurse 4)

‘And when they are doing a group we say they can stop doing it any time, they can stop attending, it’s their choice, whether they want to come or not’ (Focus group) (Support worker)

When being persuaded or directed to go for interventions was discussed in joint interviews, by family members, the people with dementia said they had enjoyed the experience and would be happy to go again. However, it is possible a person with dementia may not have felt able to express an alternative view within joint interviews.

## Discussion

This study identified influences on uptake of psychosocial interventions by exploring perspectives from people with early dementia, family members and staff. Solo and joint interviews were held with people with early dementia. Staff were also interviewed or participated in a focus group. This study appears unique in identifying influences on uptake of psychosocial interventions offered in practice settings to people with early dementia. Intervention uptake was influenced by a complex interplay of individual, service and societal influences. How people with dementia responded to diagnosis and experienced the impact of dementia on them, what dementia services offered and the relationships between them and their family members were key to encouraging uptake.

Group interventions offered (such as CST, education sessions or choir style groups) were greatly valued by most participants affected by dementia, but this was not always the case. Such findings share some similarities to research reporting that post-diagnostic support services did not always meet individual needs and preferences (Górska et al., 2013; Innes et al., 2014). We found that some people with dementia and family members worried about mixing with others with dementia, preferred solo pursuits or did not want to share their experience of dementia with others. Staff accounts also suggested that people with dementia may decline groups for similar reasons. There is much research now suggesting that tailored interventions offer a way to address individual needs (e.g. Clare et al., 2019; Gitlin et al., 2018 and Graff et al., 2006). Yet, the people with dementia and family members in this study did not describe being offered personalised interventions, although some staff described occupational therapy or cognitive rehabilitation being offered. Theme 2 (intervention appeal and perceived benefit) highlighted how much people with dementia and family members valued their community-based, non-dementia-focused activities, such as pensioners’ clubs, church, day trips, socialising with friends and family or looking after grandchildren. Although staff did not talk specifically about trying to offer interventions that may appeal to people’s personal interest, staff did acknowledge how people with dementia and family members could have busy lives, with roles and responsibilities preventing intervention uptake. Staff focused more on their experiences of offering the group interventions available within their services, although a few talked about how cognitive rehabilitation offered an opportunity to work on people with dementia and family members’ shared goals.

Self-awareness, adjusting to diagnosis and stigma were all identified as influences affecting uptake in our study. A few intervention studies also report limited awareness or difficulty adjusting as reasons for declining or drop-out (Orgeta et al., 2015; Woods et al., 2016). Yet often, such studies do not discuss such issues. This may be because those struggling to adjust to diagnosis, or with apparent limited awareness of changes or challenges associated with dementia, or those feeling stigmatised are unlikely to participate in research (Bartlett et al., 2018). However, research about awareness in early stage Alzheimer’s (Clare et al., 2012) and stigma (Burgener et al., 2015) indicates these lived experiences are important, and, we would suggest, are likely to influence uptake of interventions offered by services.

We found family members providing reassurance and encouragement to people with dementia to try new interventions was key. We also found that a few family members felt the need to be directive, even if the person with dementia was not keen to participate in an intervention offered, because the family member believed doing so may be beneficial. This nuanced sense of family support is rarely raised in research reporting interventions, with a few exceptions. For example, Milders et al. (2013) have reported some carers found it difficult or stressful to engage in activities required by a CST intervention as a reason for drop-out; whether responsibilities placed on carers were too burdensome, perhaps influencing drop outs from a group reminiscence intervention (Woods et al., 2016) or an individual CST intervention (Orgeta et al., 2015) have also been discussed. Having no suitable carer to participate alongside a person with dementia can also be a reason for non-participation in dementia research (Bartlett et al., 2018). Our findings suggest it may also be a reason for declining interventions offered in practice. We found that many of the people with dementia interviewed relied on family to take them to interventions, and staff were concerned that people who did not have family to support them were discouraged from attending interventions. Transport or location has been identified as influences on intervention experience or acceptability (Górska et al., 2013; Innes et al., 2014; Mountain & Craig, 2012). This leads to questions about how people with dementia living without family support manage to attend interventions or services they wish to.

## Limitations

Convenience samples were necessary given limited resources. Purposive sampling and recruitment until data saturation may have enhanced transferability of findings, particularly to those with different backgrounds or other settings. However, the convenience sample obtained contained some variation, in terms of types of dementia, caring relationships and the staff sample broadly reflected teams that tend to work in dementia services. Sampling people with dementia who declined interventions would have enhanced findings but would require purposive sampling from alternative or additional recruitment sources other than those we recruited from and over a longer time frame than was possible for this study. Whilst we aimed to represent the perspectives of people with dementia, most were interviewed jointly with family members. Despite attempts to support people with dementia express their views in joint interviews and analysis examining the different views expressed within joint interviews, we recognise that in some joint interviews, family member accounts dominated. However, joint interviews allowed people with dementia, who wanted the support of another person in interview, to participate. Additionally, some participants may not have recalled all interventions they had been offered or did not talk about them during interview.

## Implications

When delivering interventions in practice or research, identifying key characteristics (e.g. age, gender, caring relationships/living situation, postcode, ethnicity, diagnosis type and sexuality) about those who accept or decline could help identify underserved populations and areas for research or practice development. Considering location and ease of travel appears important to facilitating uptake. Involving people affected by early dementia in service and intervention development is needed (The Dementia Engagement and Empowerment Project, 2016) to help increase the likelihood that interventions offered are wanted and accepted. Developing inclusive communities that support and enable people with early dementia to participate in everyday life is recognised as vital (e.g. Shakespeare et al., 2019). Our findings uniquely highlight the importance of considering the intervention needs of people specifically with early dementia and suggest examination of the types of services (e.g. dementia-specific, NHS services and/or non-dementia-specific community-based services) which may be best placed to offer and provide interventions for people with early dementia is needed. Findings also suggested stigma can inhibit intervention uptake; thus, more research about how to destigmatise dementia in practice and research is needed. The impact of awareness and adjustment on uptake and engagement in interventions also merits further research. Examining how practitioners and family members support people with early dementia demonstrating apparent limited awareness could identify good practice. Ethnographic methods may further enhance understanding of intervention uptake, for example, by exploring interaction around intervention offers and responses in service settings. Also, interviewing people more than once could facilitate examination of what influences responses to intervention offers to change over time.

## Conclusion

Individual, service and societal influences interact to affect uptake of psychosocial interventions by people with early dementia. Further research examining uptake of specific interventions commonly offered to those with early dementia is needed. How interventions and which services should enable people with early dementia remain engaged in their everyday lives needs consideration. Involving people with early dementia in designing interventions aiming to support them is paramount.

## Supplemental Material

sj-pdf-1-dem-10.1177_14713012211007397 – Supplemental Material for What influences uptake of psychosocial interventions by people living with early dementia? A qualitative study Click here for additional data file. Supplemental Material, sj-pdf-1-dem-10.1177_14713012211007397 for What influences uptake of psychosocial interventions by people living with early dementia? A qualitative study by Becky Field, Elizabeth Coates and Gail Mountain in Dementia
