# Supplementary material for: What influences uptake of psychosocial interventions by people living with early dementia? A qualitative study
Source: Dementia (London). 2021 May 6;20(8):2668–88. doi: 10.1177/14713012211007397 (PMC8723173; doi:10.1177/14713012211007397)
Supplement: sj-pdf-1-dem-10.1177_14713012211007397 – Supplemental Material for What influences uptake of psychosocial interventions by people living with early dementia? A qualitative study [file sj-pdf-1-dem-10.1177_14713012211007397.pdf]

Supplemental material for article: 'What influences uptake of psychosocial interventions by people living with early dementia? A qualitative study' by Becky Field, Elizabeth Coates and Gail Mountain, in Dementia.

The following tables present key themes and subthemes identified from the qualitative thematic analysis (Braun and Clarke, 2006) of transcripts from the solo and joint interviews conducted with people with dementia and family members (one data set) and interviews the focus group completed with staff (another data set). After the key themes and subthemes from these different data sets were identified, overarching themes as presented in the main article were identified, using a process of triangulation (Farmer et al 2006).

Supplementary Table 1: Final key themes and subthemes identified from thematic analysis of transcripts from the solo and joint interviews conducted with people with dementia and family members

**Final key themes and subthemes**	
**Theme 1**	**Adjusting to life after a diagnosis**
Subtheme	*Self-awareness and differing accounts of dementia*
**Theme 2**	**Appeal of interventions and perception of benefit**
Subtheme	*Personal narratives*
Subtheme	*Mixing with others with dementia*
**Theme 3**	**The service context**
Subtheme	*Signposting*
Subtheme	*Practicalities: timing, location, travel and venues*
**Theme 4**	**Relationships**
Subtheme	*Encouragement and persuasion*
Subtheme	*Managing fear and anxiety*

Supplementary Table 2: Final key themes and subthemes identified from thematic analysis of staff interview and focus group transcripts

**Final key themes and subthemes**	
**Theme 1**	**Context: service contexts and wider society**
Subtheme	*Different types intervention to encourage engagement with services*
Subtheme	*Accessing interventions and practicalities*
Subtheme	*Sociocultural influences*
**Theme 2**	**Individual characteristics**
Subtheme	*Impacts of dementia on individuals*
Subtheme	*Individual personality and personal background*
Subtheme	*Pivotal influence of family members*
**Theme 3**	**Communication and relationships**
Subtheme	*Respecting personal choice and consent*
